# Supplementary material for: Role and effects of zinc supplementation in HIV-infected patients with immunovirological discordance: A randomized, double blind, case control study
Source: PLoS One. 2021 Jan 22;16(1):e0244823. doi: 10.1371/journal.pone.0244823 (PMC7822263; doi:10.1371/journal.pone.0244823)
Supplement: Attachment — Submitted filename: Response to Reviewers.docx [file pone.0244823.s002.docx]

*PLOS ONE* journal

Editorial Board

November 28, 2020

We thank the reviewers for their critical assessment of our manuscript. In the following we address their concerns point by point.

**Reviewer 1**

**Point 1.1**: “I find that a major bias in the patients population consists in the significant difference in the time on ART between IVD and controls, with IVD being on ART for significantly lower time. Lower ART time would mean lower possibility to achieve full immune recovery. The authors should either enroll a 3rd cohort of IVD with equal time on ART, or at least acknowledge this bias.”

R: We recognize de bias since the longer time of ART exposure, the greater immune recovery achieved, especially during the first 5 years. For this reason, we included a comparative analysis of immune recovery based on final CD4 cell count for both groups (IVD and controls). We found no differences on the median CD4 cell count at the end of the study (IVD: 39 cell/mm^3^, controls: 19 cell/mm^3^; p 0,1433). Furthermore, we do not know if hypozinchemia observed in the IVD group could be related to the time of ART exposure. This aspect was incorporated on the discussion.

**Point 1.2**: “Can the authors provide also data on HCV co-infection, which has been described to associate to low immune recovery on ART”.

R: We added the HCV frequency for both groups on table 1 (IVD: 2.9%; controls: 2.8%; p 0.739)

**Reviewer 2**

**Point 2.1**: “The investigators, however, did not provide sample size calculation and as they note, there may not have been sufficient number of study participants to show significant difference between the groups”

R: We calculated the sample size needed to assess whether the relative risk of a CD4 cell count increase above 30%. We were based on the percentages of CD4 cell increase greater than 30% from de study of Asdamongkol et al (46.1% in patients with zinc and 23.53% in patients with placebo), with a power of 80% and a confidence level of 95%. According to this calculation, a sample size of 28 patients per group was required. The calculation was made with nMaster 2.0 software.

Power was calculated for the comparison of the increasing average CD4cell count between cases (zinc) and controls (placebo), obtaining a value of 100%. This result

indicates that, although we did not reach the previously calculated sample size (28

patients per group of zinc and placebo), the sample size obtained was sufficient to assess whether the increase in CD4 cell levels is different between cases and controls.

This item is added on Methods.

**Point 2.2** : “the manuscript does not show the HIV viral load of the patients and it is not clear if the IVD patients were virally suppressed. This may play a role in the incomplete immune recovery”

R: All patients had to be virologically suppress to enter the study. This item is added on table 1 and reinforced on discussion.

**Point 2.3** “This contrasts with previous studies showing a positive effect of zinc supplementation on CD4 cell count of patients with low plasma zinc levels at baseline. While there were participants with very low zinc levels in this study, there were too few of them to make a judgement about the effectiveness of zinc supplementation in patients with low levels of zinc”

R: The study of *Asdamongko*l is the only one that has demonstrated a benefit of zinc supplementation on hypozinchemic patients and also included a low number of patients (hypozinchemia:12 (5 supplemented), normozinchemia:18 (8 supplemented) Their findings were very similar to ours (hypozinchemia:12 (6 supplemented); normozinchemia: 25 (13 supplemented) , but our *p* value was not satistically significant. The table in the article is above:

*Asdamongkol N, Phanachet P, Sungkanoparph S. Low plasma zinc levels and immunological responses to zinc supplementation in HIV‐in- fected patients with immunological discordance after antiretroviral ther- apy. J Int AIDS Soc. 2012;15 Suppl 4:152‐3.*

Our findings:

Table 4. Response of CD4 of supplemented patients accord to zinc level.

(12 hypozinchemia: 6 supplemented; 25 normozinchemia 13 supplemented)

| CD4^+^ (cells/mm^3^) ^*^ | Hypozinchemia(n=6) | Normozinchemia(n=13) | P- value |
| --- | --- | --- | --- |
| **Baseline** | 188.5 (179-224) | 193 (163.5-213) | 0.543 |
| Final | 235 (191-256) | 222.5 (206.5-261) | 0.752 |
| Δ CD4^+^ | 56 (11-59) | 31.5 (10-93.5) | 0.752 |

Futhermore, on a recent review neither *Baum, Bobat, Green, Range* or *Reich* found differences on increase of CD4 cell count after zinc supplementation at any zinc blood level. **(***AIDS Rev. 2020;22:1-10)* This aspect is added on the discussion and reference at bibliography.

**Point 2.4**: “another problem is that it is not clear whether the data was analyzed with intent to treat analyses”

R: the data was analyzed with intent to treat. This item is reinforced on the Methods.

**Point 2.5**” It also does not appear that the analyses controlled for baseline CD4 cell count which was significantly different between the groups. There is no table showing differences between the treatment and the placebo groups at the end of the study among all participants that started the study, only those with incomplete immune recovery”

R: we added on table 1 the immune recovery at the end of the study of IVD and control patients showing no differences. This aspect was reinforced on the discussion.

Thank you for your consideration of this manuscript.

Kind regards,

Macarena Silva, MD

Infectious Diseases

Hospital San Borja Arriarán

University of Chile School of Medicine

Santiago, Chile
